# The role of conspicuity in preventing bicycle crashes involving a motor vehicle

**DOI:** 10.1093/eurpub/cku117

**Published:** 2014-08-01

**Authors:** Sandar Tin Tin, Alistair Woodward, Shanthi Ameratunga

**Affiliations:** Section of Epidemiology and Biostatistics, School of Population Health, University of Auckland, Auckland, New Zealand

## Abstract

**Background:** Bicycle use, despite its proven health and other benefits, is rarely part of everyday travel for many people due to the perceived risk of injury from collision crashes. This article investigated the role of physical vs. attention conspicuity in preventing bicycle crashes involving a motor vehicle in New Zealand. **Methods:** The Taupo Bicycle Study involved 2590 adult cyclists recruited in 2006 (43.1% response rate) and followed for bicycle crash outcomes through linkage to four national databases. A composite measure of physical conspicuity was created using latent class analysis based on the use of fluorescent colours, lights and reflective materials, and the main colour of top, helmet and bike frame. Attention conspicuity was assessed based on regional differences in travel patterns and the amount of riding in a bunch. Cox regression modelling for repeated events was performed with multivariate adjustments. **Results:** During a median follow-up period of 6.4 years, 162 participants experienced 187 bicycle–motor vehicle crashes. The crash risk was not predicted by the four latent classes identified and the amount of bunch riding but was higher in Auckland, the region with the lowest level of bicycle use relative to car use. In subgroup analyses, compared to other latent classes, the most physically conspicuous group had a higher risk in Auckland but a lower risk in other regions. **Conclusion:** Conspicuity aids may not be effective in preventing bicycle–motor vehicle crashes in New Zealand, particularly in Auckland, where attention conspicuity is low.

## Introduction

About one-third of the world’s adult population is not sufficiently active partly due to an increase in the use of ‘passive’ modes of transport.^1^ Bicycle use, if integrated into daily life, provides health, environmental and economic benefits;^2^^,^^3^ yet, it is rarely part of everyday travel in many countries.^4^^,^^5^ One of the major deterrents to engaging in such activity is fear of motorized traffic.^6^

According to police reports in New Zealand (2007–2011), an average of 10 cyclists are killed and 817 are injured in collisions with a motor vehicle each year.^7^ Collision crashes often result from the driver’s failure to detect the cyclist who has the right-of-way^8^ and are termed the ‘looked-but-failed-to-see’ phenomenon.^9^^,^^10^ Features contributing to drivers failing to see a bicycle on the road include the relative rarity of cyclists, small size, slow speed and low level of perceived threat,^10^^,^^11^ suggesting that increasing the conspicuity of cyclists may reduce the risk of collisions.

Conspicuity can be classified into: physical conspicuity (distinction of an object due to its physical characteristics) and attention conspicuity (distinction of an object based on the observer’s interest and experience).^12^^,^^13^ The former may be enhanced by using conspicuity aids such as fluorescent materials, lights and reflectors. Such measures improve drivers’ detection and recognition time in experimental settings^14^ but their effect on cyclist safety is inconclusive.^15–18^ The attention conspicuity, on the other hand, may be improved by creating a more balanced transport mix. The ‘safety in numbers’ effect suggests that if more people cycle and less drive, cyclists will be safer as drivers are more likely to pay attention to the presence of cyclists.^19^^,^^20^ However, riding in a bunch may provoke negative interactions with other road users, e.g. if cyclists spread across the road.^21^

The Taupo Bicycle Study is a prospective cohort study designed to examine factors associated with regular cycling and injury risk. Our previous analysis of the study data shows slight reduction in the risk of collision crashes by using fluorescent colours, lights and reflective materials.^22^ The risk is higher in the region with the lowest level of bicycle use relative to car use but bunch riding slightly increases the risk. Limitations of the analysis, however, include limited study power due to a small number of events and inability to categorize crashes by lighting condition as this information was not usually available in the crash outcome data collected through record linkage.

This article attempted to address these issues by using longer-term outcome data (a median follow-up of 6.4 years cf. 4.6 years in the previous analysis) and by creating a composite measure of physical conspicuity based on a set of seven variables. The latter was accomplished by using latent class analysis (LCA). The association between the latent classes identified and the risk of crashes involving a motor vehicle was then assessed. The crash risk associated with bunch riding and region of residence was also updated using the extended follow-up data.

## Methods

### Design, setting and participants

The sampling frame comprised cyclists aged 16 years and over who enrolled online in the Lake Taupo Cycle Challenge, New Zealand’s largest mass cycling event held each November. Participants have varying degrees of cycling experience ranging from competitive sports cyclists to relative novices of all ages.

Recruitment was undertaken at the time of the 2006 event for the majority of participants, as described, in detail, elsewhere.^15^ Briefly, email invitations, containing a hyperlink to the study information page, were sent to 5653 contestants who provided their email addresses at registration for the event. Those who agreed to participate in the study were taken to the next page containing a web questionnaire and asked about demographic characteristics, general cycling activity and crash experience in the past 12 months, habitual use of fluorescent colours, lights and reflective materials with options ranging from ‘never’ to ‘always’, and main colour of the most commonly used bike, helmet and top (cycling shirt). The questionnaire was completed and submitted by 2438 cyclists (43.1% response rate). Another 190 cyclists were recruited from the 2008 event by including a short description about the study in the event newsletter. All participants were resurveyed in December 2009 using a web questionnaire containing similar questions as the baseline questionnaire. A total of 1537 participants completed the questionnaire. Supplementary Figure S1 presents the flow of study participation.

### Crash outcome data

In this analysis, all bicycle crashes that involved a motor vehicle, i.e. resulted from a collision or attempted evasion of a collision with a motor vehicle, were considered as the primary outcome. The data were collected through linkage to four administrative databases covering the period from the date of recruitment to 30 April 2013.

In New Zealand, Accident Compensation Corporation (ACC) provides personal injury cover for all residents and temporary visitors to New Zealand no matter who is at fault. The claims database is a major source of information on relatively minor injuries. Approval for record linkage was obtained from the ACC Research Ethics Committee.

The hospital discharge data contains information about in-patients and day patients discharged after a minimum stay of 3 h from all public hospitals and over 90% of private hospitals in New Zealand. The mortality data contains information about all deaths registered in the country. Diagnoses in each hospital visit and underlying causes of death are coded using the ICD-10-AM classification system. Bicycle collisions involving a motor vehicle were identified using the E codes V12-V14, V19.0-2 and V19.4-6. Readmissions were excluded.

In New Zealand, it is mandatory that any fatal or injury crash involving a collision with a motor vehicle on a public road be reported to the police. The crash analysis system data contains information on all police-reported bicycle collisions involving a motor vehicle.

For each participant, bicycle crashes identified across different databases were matched based on the date of crash allowing for a 2-day difference, so as to avoid double-counting of the same crash.

### Analyses

The study sample was restricted to 2590 participants who were resident in New Zealand at recruitment. Baseline data were complete for 2384 participants (92.1%). Missing values were computed using multiple imputation with 25 complete datasets incorporating baseline covariates and crash outcomes.

LCA was used to explore patterns of conspicuity aid use in the study population, based on a set of seven variables: using fluorescent colours while riding on the road (five categories), using functioning front lights, functioning back lights and reflective materials while riding in the dark (six categories each), main colour of the most commonly used top, helmet and bike frame (seven categories each). Note that it is compulsory for all cyclists to wear helmets on New Zealand roads. The analyses were conducted using PROC LCA^23^ and the number of classes that fit the data best was determined by (i) Bayesian information criterion (BIC) and sample size adjusted BIC (SABIC) with lower values indicating better model fit^24^; (ii) entropy with values closer to one indicating better classification of individuals (the value greater than 0.80 is deemed sufficient^25^); and (iii) distinguishability, meaningfulness and size of classes^23^. Attention conspicuity was assessed based on regional differences in travel patterns and the amount of riding in a bunch. The participants were categorized into residents of Auckland (New Zealand’s largest urban region), Wellington (New Zealand’s capital region) and others. The ratio of time spent cycling to time spent driving is 1:76 in Auckland, 1:48 in Wellington and 1:48 in New Zealand on average.^26^

Using bicycle crash data extracted through record linkage, incidence rates of repeated events were calculated using the person-years approach. The participants were censored on 30 April 2013 or date of death. Cox proportional hazards regression modelling for repeated events was performed using a counting process approach to assess hazards of crash involvement associated with patterns of using conspicuity aids (latent classes), amount of bunch riding and region of residence. Dependence among the multiple events was not significant in an *ad hoc* test^27^ and therefore was not corrected for in the models. Hazard ratios (HRs) were first adjusted for cycling exposure (i.e. time spent cycling on the road per week) and then adjusted for all other baseline covariates presented in Supplementary Table S1. Subgroup analyses estimated the effect of conspicuity aids in Auckland and other regions. Sensitivity analyses were undertaken by restricting the main outcome to collision crashes only. All analyses were performed using SAS (release 9.2, SAS Institute Inc., Cary, North Carolina).

To assess the impact of changes in cycling exposure and use of conspicuity aids during follow-up on the association estimates, latent transition analysis (LTA) was first performed using PROC LTA^28^ and patterns of using conspicuity aids at two time points (at baseline and in 2009) were examined. These together with repeated measurements of cycling related covariates were then incorporated in the Cox models. This analysis was restricted to 1526 cyclists who were resident in New Zealand and completed the second questionnaire.

## Results

Of the 2590 study participants, 35.5% resided in Auckland, 20.6% in Wellington and 42.5% in other regions (Supplementary Table S1). On average, the participants cycled 5 h a week on the road, of which 20% was for riding in a bunch. About 29.3% reported always wearing fluorescent colours while cycling. Of the 1731 participants who reported cycling in the dark, 83.8% always used functioning front lights, 89.9% always used functioning back lights and 49.1% always used reflective materials. The participants most commonly wore yellow or orange top (43.8%) and blue (30.8%), red (20.1%) or silver (18.2%) helmet, and rode blue (23.5%) or black (23.4%) bike.

Latent class models were estimated for one- to six-class solutions (Table 1). The four-class solution had a lower BIC and SABIC compared to others and also had a relatively high entropy (0.88), and was chosen as the best fit. Based on the conditional item probabilities (Table 2), class one was termed ‘usually conspicuous day & night’; class two was termed ‘often conspicuous during the day and do not cycle in the dark’; class three was termed ‘occasionally conspicuous day & night’; and class four was termed ‘rarely conspicuous during the day but conspicuous in the dark’.

**Table 1:** Fit indices for latent class analysis

	Classes					
	1	2	3	4	5	6
Log likelihood G-squared statistic	10020.29	9059.75	8365.10	7838.73	7681.88	7494.47
Akaike Information Criterion (AIC)	10094.29	9209.75	8591.10	8140.73	8059.88	7948.47
Consistent AIC (CAIC)	10328.44	9724.00	9365.91	9176.09	9355.79	9504.95
Schwarz Bayesian Information Criterion (BIC)	10291.44	9649.00	9252.91	9025.09	9166.79	9277.95
Sample size adjusted BIC (SABIC)	10173.90	9410.71	8893.87	8545.33	8566.29	8556.70
Entropy	1.00	1.00	0.84	0.88	0.85	0.85

**Table 2:** Item response probabilities (SE) by latent class membership

Variables	Latent classes			
	One	Two	Three	Four
	*N* = 929	*N* = 903	*N* = 216	*N* = 721
	33.1%	33.0%	8.0%	25.8%
Total	0.34 (0.02)	0.33 (0.01)	0.08 (0.01)	0.25 (0.02)
Wear fluorescent colours				
0% (never)	0.01 (0.01)	0.22 (0.01)	0.23 (0.03)	0.41 (0.03)
25%	0.06 (0.01)	0.15 (0.01)	0.16 (0.03)	0.25 (0.02)
50%	0.17 (0.02)	0.14 (0.01)	0.15 (0.03)	0.27 (0.02)
75%	0.28 (0.02)	0.16 (0.01)	0.21 (0.03)	0.08 (0.02)
100% (always)	0.49 (0.03)	0.33 (0.02)	0.25 (0.03)	0.00 (0.01)
Use front lights in the dark				
0% (never)	0.03 (0.01)	0.00 (0.00)	0.55 (0.04)	0.00 (0.01)
25%	0.00 (0.00)	0.00 (0.00)	0.14 (0.02)	0.00 (0.00)
50%	0.00 (0.00)	0.00 (0.00)	0.05 (0.02)	0.02 (0.01)
75%	0.01 (0.01)	0.00 (0.00)	0.22 (0.03)	0.05 (0.01)
100% (always)	0.96 (0.01)	0.00 (0.00)	0.04 (0.02)	0.93 (0.02)
Do not cycle in the dark	0.00 (0.00)	1.00 (0.00)	0.00 (0.00)	0.00 (0.00)
Use back lights in the dark				
0% (never)	0.00 (0.00)	0.00 (0.00)	0.34 (0.04)	0.00 (0.00)
25%	0.00 (0.00)	0.00 (0.00)	0.13 (0.02)	0.00 (0.00)
50%	0.00 (0.00)	0.00 (0.00)	0.05 (0.02)	0.00 (0.00)
75%	0.00 (0.00)	0.00 (0.00)	0.23 (0.03)	0.01 (0.01)
100% (always)	1.00 (0.00)	0.00 (0.00)	0.25 (0.05)	0.98 (0.01)
Do not cycle in the dark	0.00 (0.00)	1.00 (0.00)	0.00 (0.00)	0.00 (0.00)
Use reflective materials in the dark				
0% (never)	0.10 (0.01)	0.00 (0.00)	0.39 (0.04)	0.33 (0.02)
25%	0.04 (0.01)	0.00 (0.00)	0.15 (0.03)	0.16 (0.02)
50%	0.04 (0.01)	0.00 (0.00)	0.11 (0.02)	0.13 (0.02)
75%	0.08 (0.01)	0.00 (0.00)	0.08 (0.02)	0.14 (0.02)
100% (always)	0.74 (0.02)	0.00 (0.00)	0.26 (0.04)	0.24 (0.03)
Do not cycle in the dark	0.00 (0.00)	1.00 (0.00)	0.00 (0.00)	0.00 (0.00)
Main top colour				
Black	0.01 (0.00)	0.02 (0.00)	0.05 (0.02)	0.07 (0.01)
Blue	0.09 (0.01)	0.17 (0.01)	0.24 (0.03)	0.32 (0.02)
Red	0.10 (0.01)	0.15 (0.01)	0.12 (0.02)	0.16 (0.02)
Yellow or orange	0.63 (0.02)	0.46 (0.02)	0.43 (0.04)	0.17 (0.03)
Silver	0.01 (0.00)	0.01 (0.00)	0.01 (0.01)	0.02 (0.01)
White	0.05 (0.01)	0.08 (0.01)	0.08 (0.02)	0.15 (0.02)
Others	0.11 (0.01)	0.10 (0.01)	0.07 (0.02)	0.10 (0.01)
Main helmet colour				
Black	0.09 (0.01)	0.11 (0.01)	0.09 (0.02)	0.10 (0.01)
Blue	0.31 (0.02)	0.33 (0.02)	0.33 (0.03)	0.28 (0.02)
Red	0.20 (0.02)	0.19 (0.01)	0.22 (0.03)	0.20 (0.02)
Yellow or orange	0.06 (0.01)	0.05 (0.01)	0.08 (0.02)	0.05 (0.01)
Silver	0.19 (0.01)	0.19 (0.01)	0.16 (0.03)	0.17 (0.02)
White	0.09 (0.01)	0.08 (0.01)	0.09 (0.02)	0.13 (0.02)
Others	0.05 (0.01)	0.06 (0.01)	0.04 (0.01)	0.06 (0.01)
Main bike colour				
Black	0.26 (0.02)	0.21 (0.01)	0.21 (0.03)	0.24 (0.02)
Blue	0.24 (0.02)	0.25 (0.01)	0.26 (0.03)	0.21 (0.02)
Red	0.15 (0.01)	0.16 (0.01)	0.19 (0.03)	0.18 (0.02)
Yellow or orange	0.07 (0.01)	0.05 (0.01)	0.04 (0.01)	0.05 (0.01)
Silver	0.17 (0.01)	0.16 (0.01)	0.13 (0.02)	0.17 (0.02)
White	0.08 (0.01)	0.10 (0.01)	0.12 (0.02)	0.08 (0.01)
Others	0.04 (0.01)	0.06 (0.01)	0.06 (0.02)	0.06 (0.01)

During a median follow-up of 6.4 years, 162 participants experienced 187 bicycle crashes involving a motor vehicle (19 experienced 2 crashes and 3 experienced 3 crashes), corresponding to 12 crashes (95% CI: 9.91, 13.28) per 1000 person-years (Table 3). The crash risk was lower in class three but was similar across the remaining classes especially when demographics and cycling-related covariates were adjusted for. The risk was higher in Auckland but was not predicted by bunch riding. In the subgroup analysis, compared with other classes, class one had a higher crash risk in Auckland but a lower risk in other regions (Table 4). The results were similar when the main outcome was restricted to collision crashes only.

**Table 3:** Hazard ratio estimates for bicycle crashes involving a motor vehicle

	No. of crashes	Person-years	Rate per 1000 person-years	Crude HR (95% CI)	Adjusted HR (95% CI)^a^	Adjusted HR (95% CI)^b^
Total	187	16 249.68	11.51 (9.91, 13.28)			
Conspicuity aid use						
Class one	73	5351.58	13.64 (10.69, 17.15)	1.07 (0.75, 1.52)	1.08 (0.76, 1.55)	1.03 (0.68, 1.54)
Class two	50	5306.77	9.42 (6.99, 12.42)	0.74 (0.50, 1.09)	0.86 (0.58, 1.27)	1.09 (0.70, 1.69)
Class three	9	1313.13	6.85 (3.13, 13.01)	0.54 (0.27, 1.11)	0.60 (0.29, 1.22)	0.66 (0.52, 1.06)
Class four	54	4233.20	12.76 (9.58, 16.64)	1.00	1.00	1.00
Missing	1	45.02	22.21 (0.56, 123.76)			
% riding in a bunch				1.008 (1.003, 1.013)	1.007 (1.002, 1.012)	1.004 (0.999, 1.010)
Ever ride in a bunch						
Yes	144	11 518.50	12.50 (10.54, 14.72)	1.44 (1.01, 2.06)	1.27 (0.89, 1.82)	1.01 (0.70, 1.47)
No	40	4604.57	8.69 (6.21, 11.83)	1.00	1.00	1.00
Missing	3	126.61	23.70 (4.89, 69.25)			
Region of residence						
Auckland	88	5725.97	15.37 (12.33, 18.93)	1.00	1.00	1.00
Wellington	36	3372.39	10.67 (7.48, 14.78)	0.70 (0.47, 1.03)	0.74 (0.50, 1.09)	0.78 (0.52, 1.16)
Others	63	6937.94	9.08 (6.98, 11.62)	0.59 (0.43, 0.82)	0.59 (0.42, 0.82)	0.74 (0.52, 1.06)
Missing	0	213.39	0.00			

a: Adjusted for hours spent cycling on the road per week.

b: Adjusted for all baseline covariates.

**Table 4:** Associations between conspicuity aid use and bicycle crashes involving a motor vehicle in Auckland vs. other regions

	Adjusted HR (95% CI)^a^	
	Auckland	Others
Conspicuity aid use		
Class one	1.43 (0.78, 2.64)	0.77 (0.43, 1.38)
Class two	1.09 (0.54, 2.23)	1.04 (0.58, 1.89)
Class three	0.60 (0.18, 2.02)	0.72 (0.29, 1.83)
Class four	1.00	1.00

a: Adjusted for all baseline covariates.

Latent transition analyses showed that the chance of being in the same class at baseline and follow-up ranged between 49% (class one) and 93% (class three) (Supplementary Table S2). Changes in latent membership status as well as other cycling related variables at the two time points did not substantially change the effect estimates (Supplementary Table S3).

## Discussion

### Main findings

In this study, cyclists experienced 12 crashes involving a motor vehicle per 1000 person-years. The crash risk was similar across different patterns of using conspicuity aids except that the *‘*occasionally conspicuous day & night’ group had a lower risk relative to others. The crash risk was higher and conspicuity aids were less likely to be effective in Auckland compared with the rest of New Zealand.

### Strengths and limitations

This article represents the first attempt to identify distinct patterns of conspicuity aid use in a cohort of cyclists using LCA. Baseline data were near-complete as mandatory fields and validation checks were incorporated in the web questionnaire. Crash outcome data were collected from four administrative databases, thereby minimising potential biases associated with loss to follow-up and self-reports.

Ascertainment of crash outcomes, however, may be affected by personal, social and health service factors as well as the quality of individual data sources and record linkage. In our previous analysis, the linked data was 83.3% complete for collision crashes.^29^ As the data did not contain detailed information on the circumstances of the crash, we attempted to account for lighting condition at the time of the crash by creating composite patterns of using conspicuity aids but were not able to account for road and traffic conditions. Although longer-term outcome data were used in this analysis, the study power may still be limited due to relatively rare crash events. Self-reported cycling exposure and use of conspicuity aids may not be accurate and may change over time. Nevertheless, potential misclassifications of crash outcome and baseline data tend to be non-differential in a prospective cohort study.^30^ Moreover, in the sub-group of participants where baseline information was re-assessed, changes in patterns of using conspicuity aids and cycling-related covariates did not substantially change the association estimates. Finally, our participants cannot be considered representative of all New Zealand cyclists; however, this may have minimal impact on the association estimates.^31^ Importantly, the participants represented a wide variation with regard to demographics, cycling exposure and experience and the results will be valid for all cyclists and traffic environments similar to New Zealand.

### Interpretation

A Cochrane review of 42 trials (updated in 2009) concluded that fluorescent materials improve drivers’ detection and recognition of cyclists and pedestrians in the day-time and lamps, flashing lights and retroreflective materials have similar effect in the night-time.^14^ As it was not possible to categorize crashes by lighting condition, this analysis used a composite measure of conspicuity and found no significant association with the risk of crashes involving a motor vehicle. Likewise in a previous cohort study involving bicycle commuters in Portland, using lights in the dark or reflective materials did not predict the risk of traumatic events (defined as a cycling event leading to injury).^16^ A strong protective effect of fluorescent colours observed in our earlier (cross-sectional) analysis^15^ may be due to failure to exclude cyclist only crashes.

Our study is one of very few examining the effect of cyclist conspicuity on incident crashes, but the design did not allow us to account for behaviours of involved parties and road and traffic conditions before the crash. Some case–control studies attempted to address this issue by measuring cyclists’ acute behaviour including use of conspicuity aids before a crash. A Canadian study observed that the risk of collisions with a motor vehicle was increased by wearing fluorescent clothing but decreased by wearing white or coloured clothing.^17^ Likewise, a UK study reported an increased risk of collision or evasion crashes by using any item of fluorescent or reflective material.^18^ Additionally, a recent experiment in the UK reported little effect of fluorescent clothing on drivers’ overtaking proximities.^32^

Overall, evidence for the effectiveness of conspicuity aids in reducing bicycle crash risk remains equivocal. Some have argued that cyclists’ misconceptions about their conspicuity and subsequent risk compensation could play a role in minimising potential benefits. In an Australian study, cyclists overestimated their night-time visibility and occasional cyclists were more likely than frequent cyclists to do so.^33^^,^^34^ There were also misjudgements on the conspicuity benefits of fluorescent vs. retroreflective materials at night. If cyclists using conspicuity aids are confident of being seen, they may be engaged in compensatory behaviour changes, e.g. cycling in more dangerous circumstances.^18^

Additionally, expectation and awareness of drivers may need to be considered in the ‘looked-but-failed-to-see’ errors. Previous research on pedestrian conspicuity demonstrated an increase in detection distance with expectancy.^35^ This may explain why conspicuity aids appear to be more effective in simulated settings where test observers were primed to expect the target. In real driving conditions, however, drivers develop a visual search strategy which focuses on frequent, major dangers but filters out infrequent objects.^11^ In car-dominated environments, bicycles are relatively rare and may not attract drivers’ attention. In a recent eye tracking experiment conducted in the UK, drivers failed to see 22% of cyclists on the road, outnumbering motorcyclists (15%) and pedestrians (4%).^36^

Cyclists’ attention conspicuity may be improved if more people cycle and less drive a car. Previous research on the ‘safety in numbers’ effect associated a lower crash risk with a higher level of cycling.^19^^,^^37^ This is true in the New Zealand context particularly if the level of car use is also taken into account.^20^ This study shows similar findings with the crash risk being higher in Auckland, the region with the lowest level of bicycle use relative to car use. Additionally, in our subgroup analysis, conspicuity aids appear to be less effective in Auckland compared to other regions. However, bunch riding did not provide a protective effect possibly because cyclists are more likely to take risks^21^ and less likely to notice road hazards^38^ while riding in a group for recreation.

Our findings underscore the need for a more balanced transport mix, i.e. shifting trips from cars to active modes, to improve cyclists’ conspicuity and their safety on the road. This may also increase physical activity, improve health, reduce emissions and save fuel.^2^^,^^3^^,^^39^ While the ‘coordinated implementation of the multi-faceted, mutually reinforcing set of policies’ is required as in European countries,^40^ much could be done in the short term, e.g. reducing the speed limit in residential streets. Lower speeds may also provide drivers more time to detect cyclists as suggested in a previous study.^11^

To conclude, conspicuity aids may provide little preventive effect on bicycle crashes involving a motor vehicle in New Zealand, particularly in Auckland, where bicycle use is low relative to car use. There is a need to prioritise broader environmental strategies to promote active modes of travel and the safety of cyclists.

## Supplementary data

Supplementary data are available at *EURPUB* online.
